# A Queer Sight

**Published:** 1878-03

**Authors:** 


**A QUEER SIGHT.**—Not long ago a man came into the hospital as doleful a looking object as one need to look at. There was a combination of expression in his countenance which was perfectly ludicrous. He was evidently in great pain, but he looked most particularly solemn, for he could not imagine what was the matter with himself. His arms were extended at an angle of about forty-five degrees, and hanging helplessly downwards, as if there was something on his fingers which he was anxious to keep from his trowserloons. Both his arms were dislocated downwards at the shoulder, caused by his eating too much supper! Now the blessing of eating nothing at all later than a one o'clock dinner is a "double and thribble one," at least to sedentary and otherwise inactive persons—namely, a night of luscious sleep, a glorious appetite for breakfast, and a sunny temper all day, to say nothing of exemption from horrible dreams, walking out of third-story windows, and ghostly nightmares. The man had eaten a late dinner of bacon and cabbage, and being very tired, retired immediately to bed, but not to sound repose, for he dreamed that a whole regiment of hobgoblins was after him. He put forth almost superhuman efforts to escape, but at the very instant of their laying their claws on him, he made a tremendous leap upwards, with the result of the painful luxation just named. Those who eat heartily, late in the day, do not always escape so easily, for naturally turning on their backs, the weight of what they have eaten steadily presses on the great blood-vessels of the body near the backbone, arrests the flow of blood, dams it up in the brain until effusion takes place, that is, apoplexy, and is instant death. Such are the cases where persons are found dead in their beds in the morning, with the accompanying remark: "He seemed as well yesterday as he ever was in his life."

